# Too cold for warm glow? Christmas-season effects in charitable giving

**DOI:** 10.1371/journal.pone.0215844

**Published:** 2019-05-22

**Authors:** Stephan Müller, Holger A. Rau

**Affiliations:** University of Göttingen, Göttingen, Germany; Heidelberg University, GERMANY

## Abstract

This paper analyzes seasonal effects and their potential drivers in charitable giving. We conduct two studies to analyze whether donations to the German Red Cross differ between the Christmas season and summer. In study 1 we find that in the pre-Christmas shopping season prosocial subjects almost donate 50% less compared to prosocials in summer. In study 2 we replicate the low donations in the Christmas season. In an extensive questionnaire we control for several causes of this effect. The data suggest that the higher prosocials’ self-reported stress level, the lower the donations. The higher their relative savings, the lower the giving. Our questionnaire rules out that “donation fatigue” matters. That is, donations do not depend on the number of charitable campaigns subjects are confronted with and their engagement in these activities during Christmas season outside the lab.

## 1 Introduction

In the United States, more than one third of annual donations (33.6%) happen in the “giving season” between Thanksgiving and Christmas. Similarly, the Center on Philanthropy [1] finds that 43% of high-income households donate more between Thanksgiving and New Year’s Day. The lion’s share can be attributed to December where 17.5% of the year’s donations are collected [2]. Likewise, most of the campaigns take place in the holiday time, an important factor, which may contribute to increased donations during the Christmas period. Data from 2017 reveal that on “Giving Tuesday” 2.4 million social media engagements happened for charitable giving purposes (see http://www.givingtuesday.org.). Cairns and Slonim [3] argue that donations in churches are higher at Easter and at Christmas. However, a possible reason for the increase in donations may be that more people go to church during holidays. In this vein, Greenberg [4] finds that during the holiday season tipping rates in restaurants are higher.

At the same time, raising donations by means of more campaigns comes at a cost, as they are often expensive or inefficient [5]. There is evidence that fundraising spending may account for 24% of overhead costs [6], which may hinder donations if donors are informed of them [7, 8]. Moreover, fundraising campaigns may be especially costly during the Christmas period as solicitors’ compensation is higher and commercials are more expensive at this time. Hence, the optimal timing of fundraising activity may have important implications for the marketing and success of campaigns. As a key for a better understanding of the drivers of the aggregate seasonal dynamics, we address the following question: *Is it that more money is collected in the Christmas season solely, because of higher fundraising activity? Or is it that during this time people generally give more*?

In two experimental studies, this paper compares people’s willingness to give in the Christmas season and summer. Controlled donation experiments have been successfully applied in many laboratory settings [9, 10, 11] or online experiments [12, 13, 14]. Benz and Meier [15] present evidence that laboratory data of donation decisions achieves external validity. The article highlights that prosocial behavior in experiments is correlated with people’s donation decisions in the field. Moreover, a laboratory approach can counteract possible obstacles of the field. Gneezy and Rustichini [16] emphasize that the amount collected may be influenced by a solicitor’s work motivation. In field data it is possible that tax incentives may influence individual donation behavior [17]. Studying seasonal effects may be problematic, as heterogeneous effects may arise. In street solicitations subjects may be affected by the surroundings such as Christmas music playing. The studential subject pool of our experiment guarantees that participants are homogeneous. The experiments also allow us to control for individual preferences such as Social Value Orientation. This is of importance since prosociality is an explanatory factor for charitable giving [18].

The results show that the willingness to give is significantly lower in the Christmas season than in summer. During Christmas time average donations are substantially lower (19% of subjects’ endowment) than in summer (27% of subjects’ endowment). The lower donations can be explained by the behavior of prosocials who donate less often and significantly lower amounts (18%) in the Christmas season as compared to prosocial subjects in summer (35%). In a second study we analyze in a questionnaire whether two empirical particularities (higher stress levels and higher consumption levels) could be related to lower giving of prosocial subjects in winter. The data perfectly replicate the findings of our first study. The results show that prosocials who report a higher perceived degree of stress in the Christmas season compared to the rest of the year, contribute 34% less than prosocials who are not affected. We also find that the higher the subjects’ self-reported relative savings compared to the rest of the year, the lower their donations.

## Study 1: Seasonal effects in charitable giving

The objective of study 1 is to analyze whether subjects’ willingness to give differs between the Christmas season and summer. In a controlled-laboratory experiment, we analyze whether prosocials give more than individualists and whether the seasonal effect is more pronounced for prosocials.

### Method

The study concentrates on a one-factor (donations: in the Christmas season vs. summer time) experimental design. The participants were randomly assigned to the experimental sessions. The study consists of two experiments, which where conducted during summer and during the Christmas period. The summer experiment was conducted in June 2015. Here, we collected data of 72 subjects at a German university (University of Göttingen; 56% female; mean age = 23.81 years; standard deviation [SD] = 3.02; the age range was between 18 and 33). The winter experiment was conducted in the last week of November 2016 and in December 2016 at the same university. For the winter data we had 94 subjects (52% female; mean age = 23.93 years; standard deviation [SD] = 4.84; the age range was between 18 and 49. The age of one subject could not be identified, as it entered an age of “0” in the questionnaire). The summer and winter experiments were programmed in z-Tree [19]. In both experiments subjects from various fields were recruited with the data-base tool ORSEE [20]. No IRB was acquired as there is no internal review board at the university where the experiments were conducted. In accordance with the Declaration of Helsinki, all participants were requested to read an online consent form and agree with its terms (by clicking) before registering to take part in an experiment. Participants were guaranteed the anonymity of the data generated during the experiment. In each experiment subjects were informed that they participate in a study of decision making. We declare that this submission complies with the EconStore Terms of Use (https://www.econstor.eu/Nutzungsbedingungen). For the recruitment procedure there was no specific inclusion criteria. For the winter experiments we excluded subjects from recruitment who already participated in the summer experiment one year earlier. Furthermore, subjects who participated in other donation studies were not recruited. A session lasted 60 minutes. Subjects earned, on average, €12.12 including a show-up fee of €2.

The summer and winter experiments comprised the same three stages. Subjects received the instructions of each stage before it started. They were not informed of the outcome of the consecutive stages until the experiments were finished. At the end of the experiments, one stage was randomly selected to be paid out. Subjects earned Talers and the exchange rate was 10 Talers = 1 Euro. In the first stage, we elicited risk preferences with the task introduced by Gneezy and Potters [21]. We elicit this data for another study, which focuses on cooperation under uncertainty. The results of this stage were not revealed before the very end of the experiment. In the second stage of both experiments subjects received money from the experimenter. Subjects were informed that they could donate this money in integers to the German “Red Cross” [9]. We explicitly informed them that the donations would be transferred via online transactions after the experiment. In 3 of 4 winter sessions we tested whether overhead costs mattered. This data were collected for another study on cooperation under uncertainty. In this respect, subjects could click on a info button to query this information. 29 of 71 subjects did so. The distribution of the donations is not different between the three sessions where information could be obtained and the remaining one, where this was not possible (Kolmogorov-Smirnov test, *p* = .48). We merge this data for the winter sessions. To ensure credibility, subjects could stay after the experiment was finished and watch us process the online transaction. The third stage was a one-shot public good game, which is also part of the other study. The results of this public-good game were not revealed before the very end of the experiment. Afterwards, we measured the social value orientation (SVO) [22] in a non-incenticized setting. We opted for the van Lange et al. [22] set-up, as choices in this setting are by design intrinsically motivated, which is an important layer of prosocial behavior and charitable giving. Our results on subjects’ SVO show that the non-incentivized elicitation leads to the same fraction of prosocials and individualists as in the study of Grosch and Rau [23], which applied the incentivized measure of Murphy et al., [24] (Grosch and Rau, 2017 and the current study find that 66% of the subjects are prosocial and 34% are individualists. This is confirmed by a *χ*^2^-test, *p* = 0.99). In the Van Lange et al. [22] SVO-elicitation task subjects were told that they are randomly matched with another person. They knew that they and the other person decide at the same time. Subjects were told that they and the other person had to choose between three allocations of points between them and the other person. Subjects were told that the points are of value (e.g.,“Each of these points is of value. The more points you receive, the better it is for you. The more points the other person receives the better it is for her/him.”). They knew that they had to complete nine decision sets with three choices each. For each of the decision set one of the three possible answers either corresponds to a *prosocial*, *individualistic* or *competitive* choice. Subjects can be classified as one of three social types, as a result of the most often chosen answer type. The classification is possible, if subjects gave at least five times the answer, which corresponds to the same social type. Please see the on-screen instructions of the SVO test in the Appendix for a detailed overview of all nine decision sets and the coding of the answers in correspondence to the social type.

### Results

The randomization of subjects was successful between the two seasons. For details, see Table A in S1 Appendix. We classified one subject with a competitive SVO. We dropped this subject. The results do not change if we include it. In what follows, we will always report two-sided p-values.

#### Seasonal effects in charitable giving

**Fig 1:**
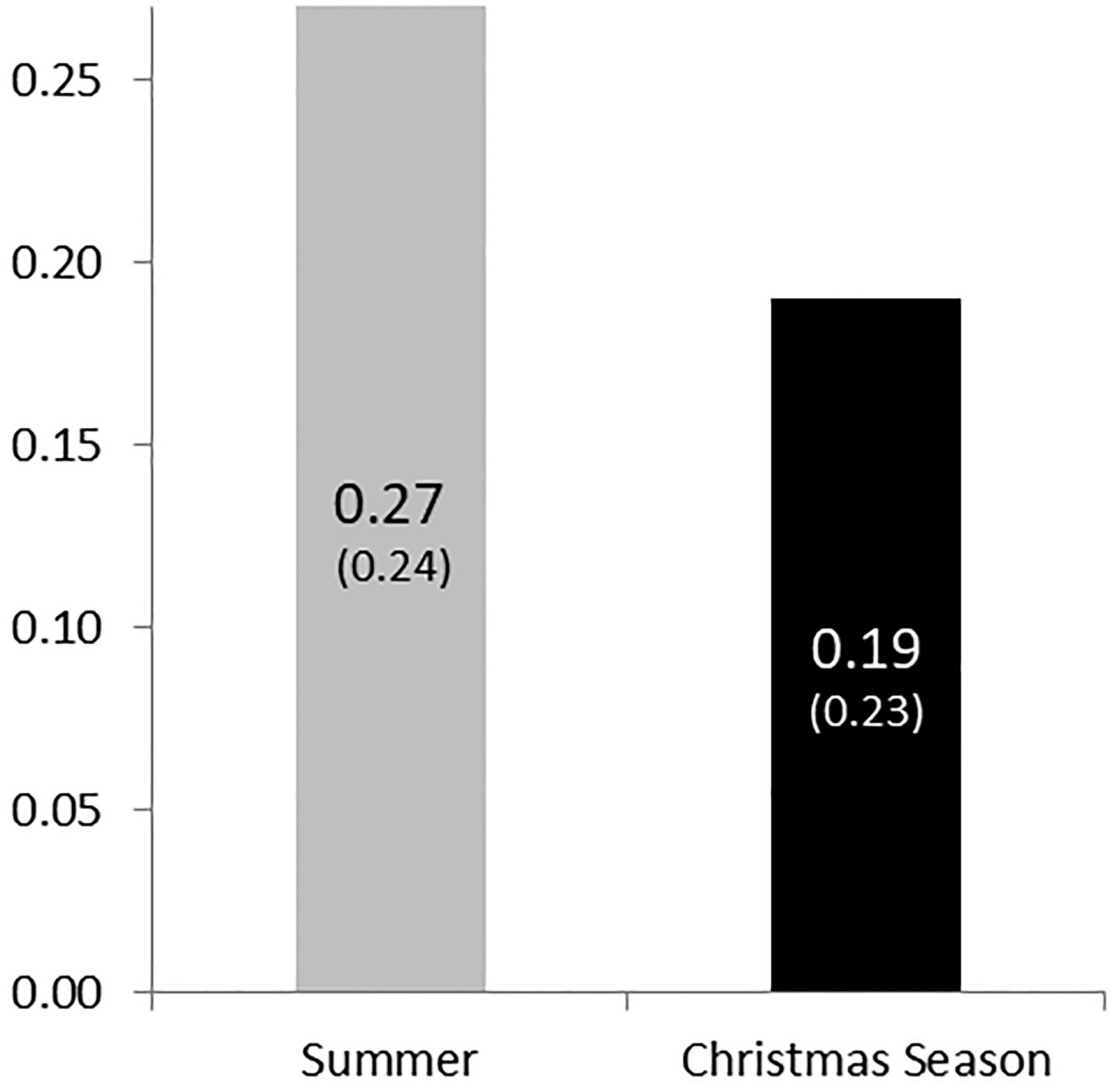
Percentage of donations across the two seasons. SD in parentheses.

Fig 1 reports our main result on seasonal effects in charitable giving. The diagram displays the average percentage of donations to the German Red Cross in summer and the Christmas season.

In summer we find that subjects donate 27%, which is similar to the results of a meta study (28%) on experimental dictator games [25]. Surprisingly, we find that donations in the Christmas season are substantially lower (19%) as compared to summer. Subjects’ willingness to give is lower by about 30%. A one-way ANOVA test reveals a significant effect of the donation season on charitable giving (*F*(1, 166) = 4.73, *p* < .05).

**Result 1**: *In the Christmas season subjects donate about 30% less than subjects in summer*.

#### Donation behavior in light of SVO

Focusing on the impact of SVO we find that prosocials donate almost 40% more than individualistic subjects. A one-way ANOVA test reveals a significant effect of subjects’ SVO type (prosocial vs. individualistic) on the donation percentage (*F*(1, 156) = 3.63, *p* = .05). This confirms the findings of Bekkers and Wiepking [17] on the effect of prosociality on charitable giving.

**Result 2a**: *Prosocial subjects donate significantly more than individualistic ones*.

**Fig 2:**
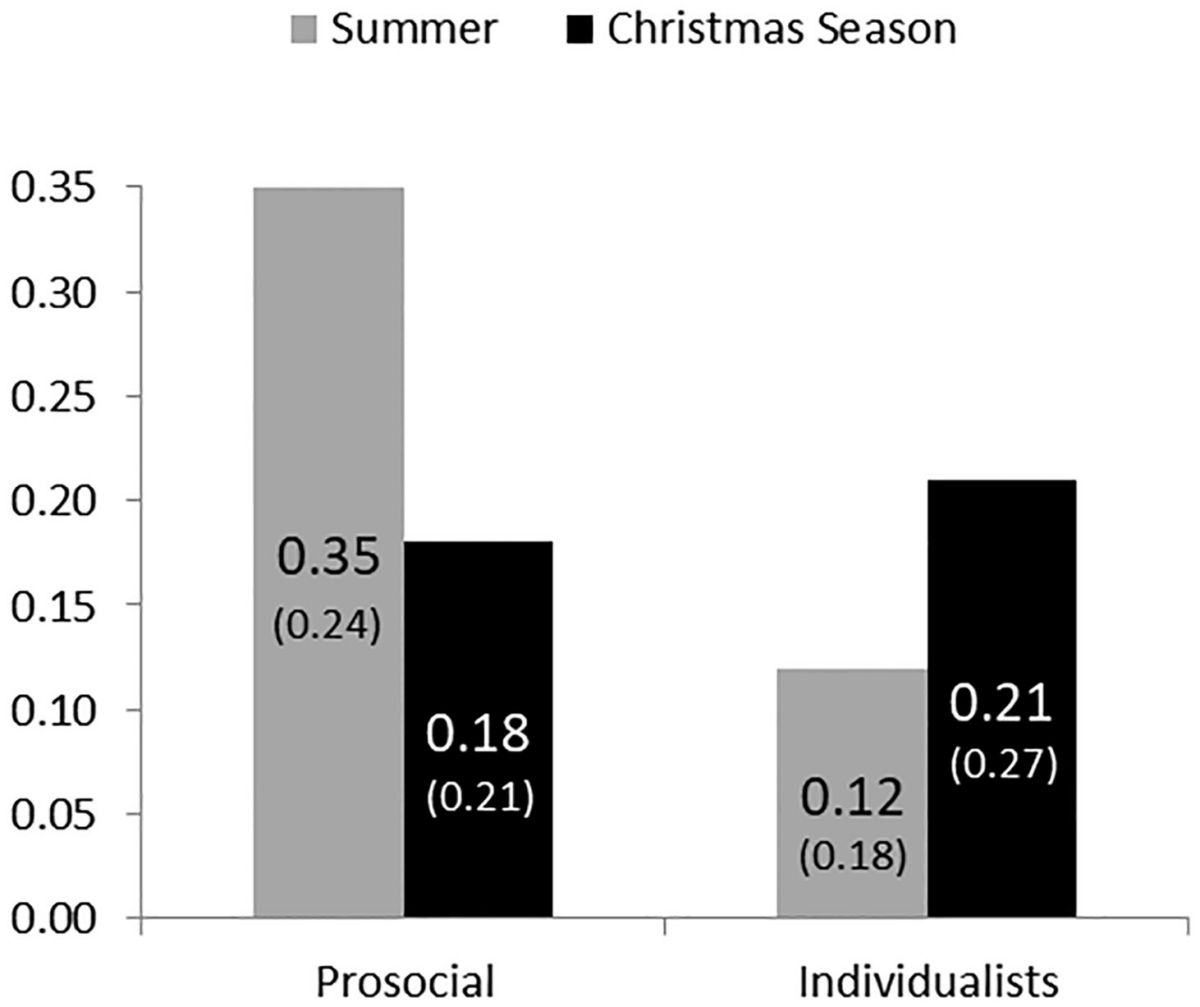
Percentage of donations conditioned on SVO. SD in parentheses.

Next, Fig 2 illustrates the percentage of donations conditioned on SVO. The diagram distinguishes between prosocial and individualistic subjects. With the SVO task we could classify 156 subjects (97 prosocials, 59 individualists). 10 subjects revealed inconsistent choices. The diagram focuses on the 156 subjects who could be classified.

The donation difference between prosocials and individualists is pronounced in summer where prosocials give substantially more (35%) than individualists (12%) (one-way ANOVA test (*F*(1, 64) = 14.63, *p* < .01). No significant difference can be found in the Christmas season when comparing the donation percentage of prosocials (18%) and individualists (21%) (one-way ANOVA test (*F*(1, 92) = .35, *p* = .55). In the Christmas season, prosocial subjects give only almost one half (18%) of the donated amount (35%) by prosocials in summer. This difference is highly significant for a one-way ANOVA test (*F*(1, 97) = 13.79, *p* < .01). Individualists donate similar levels across seasons (one-way ANOVA test (*F*(1, 59) = 1.64, *p* = .20). We conclude that the seasonal effect is driven by prosocials who show a more pronounced reaction to the Christmas season.

Next, we focus on the interaction effect of prosocial subjects and charitable giving in the Christmas season. We run a two-way analysis of variance on donation percentage and include *prosocial* (a dummy, which is positive for prosocials), *Christmas season* (a dummy, which is positive for the Christmas season), and the interaction of *prosocial* and *Christmas season*. The ANOVA test reveals significant effects for *prosocial* (*F*(1, 156) = 6.15, *p* = .01) and the interaction of *prosocial* × *Christmas season* (*F*(1, 156) = 10.49, *p* < .01) and no significant effect for the seasonal dummy (*F*(1, 156) = 1.19, *p* = .27). Hence, the seasonal effect is driven by prosocials who substantially give less during the Christmas season as compared to prosocials in summer.

Focusing on the summer data we find that donation distributions of prosocials and individualists are significantly different (Kolmogorov-Smirnov test, *p* = .01). No difference can be found in the Christmas season data (Kolmogorov-Smirnov test, *p* = .80). In the summer data, the fraction of subjects who make a zero contribution is significantly smaller for prosocial subjects (9%) than for individualistic subjects (52%) (Fisher’s exact test, *p* < .01). No differences can be found in the Christmas season data (Fisher’s exact test, *p* = .83). Moreover, we find that the fraction of prosocials, which make a zero contribution is significantly higher in the winter data (35%) compared to the summer data (9%) (Fisher’s exact test, *p* < .01). In light of the higher fraction of zero contributions among prosocials in winter, we check whether this drives the observed lower average donations. We find, however, that prosocials who donated a positive amount donate a significantly lower amount in winter (29%) as compared to prosocial donors in summer (38%) (one-way ANOVA test (*F*(1, 72) = 3.39, *p* = .07). Hence, in winter prosocials are not only less likely to give, in fact if they donate, they also give less.

**Result 2b**: *The seasonal effect is driven by prosocials who significantly give less in the Christmas season than prosocials in summer. First, prosocials are less likely to give in the Christmas season. Second, if they donate they also give less at this time*.

As a robustness check we additionally run Tobit regressions. Our estimation results confirm all of the results presented so far. We refer the reader to Table C in S1 Appendix.

## Study 2: Drivers of the lower donations

To find explanations for the surprising lower donations of prosocials in winter, we ran a second study, which took place in November 2017. We also aim to replicate the Christmas-season effect we observed in study 1. In a questionnaire we ask subjects about their perceived level of emotional stress in the Christmas season to study whether lower donations may be linked to subjects’ self-reported stress levels. The reason is that stress is associated with a lower level of empathy [26]. Empirical findings highlight that stress may be of relevance in the Christmas period, as they show an increased rate of cardiac deaths between Thanksgiving and Christmas [27]. Another factor, which may lower the sum of donations is increased consumption spending around Black Friday. The reason is that substitution effects may play a role. If subjects consume more, there is less money left, which can be used for donations. Higher consumption activity during Black Friday may in turn amplify subjects’ stress levels. This may be due to the fact that subjects’ with increased consumption activity in the Christmas season may permanently try to chase for the best deals, within a limited time horizon. Moreover, there is evidence of a dead weight loss of Christmas [28] caused by information asymmetries between the person who buys a gift and the presentee. Thus, it is likely that most people feel stressed by the search for the right presents to meet the needs and the expectations of their family. For these reasons we launched the second study in the week after Black Friday, which is similar to the timing of study 1.

### Method

Study 2 is another experiment that was run at the same German university as study 1 (University of Göttingen) in the week after Black Friday (November 2017). We collected the data of 72 subjects (58% female; mean age = 22.60 years; standard deviation [SD] = 5.00; the age range was between 18 and 50). For the recruitment procedure there was no specific inclusion criteria. For study 2 we excluded subjects from recruitment who already participated in one of the experiments of study 1. Furthermore, subjects who participated in other donation studies were not recruited. A session lasted 60 minutes. Subjects earned, on average, €11.79 including a show-up fee of €2.

The experiment was incentivized, i.e., subjects received money from the experimenter, which could be used for charitable giving. Everything was identical to study 1 where subjects could donate 100 Talers (with an exchange rate of 10 Talers = 1 Euro) to the German Red Cross. Afterwards, we again measured subjects’ SVO with the method of Van Lange [22]. The only difference of the experiment was that we added a post-experimental questionnaire. On a 5-point Likert scale we asked subjects to compare their (current) perceived stress levels to the rest of the year. We asked: “Since last month, do you perceive a higher level of stress compared to the rest of the year?” The Likert scale was defined as follows: 1 = a much lower level; 2 = a lower level; 3 = a similar level; 4 = a higher level; 5 = a much higher level. With this question we calculate a measure called *relative stress*. Additionally, subjects completed the 30-item “Perceived Stress Questionnaire (PSQ)” introduced by Levenstein [29]. Each item focuses on a stress-related question where subjects can answer based on a 4-points Likert scale. The questionnaire allows us to derive a PSQ stress index (see the instructions in the Appendix). On a 5-point Likert scale we asked subjects to compare their (current) consumption spending to the rest of the year. We asked: “Since last month, have you saved more compared to the rest of the year?” The Likert scale was defined as follows: 1 = much less; 2 = less; 3 = a similar level; 4 = more; 5 = much more. With the question we calculate a variable called *relative savings*. We also ask questions concerning consumption and saving patterns in the Christmas season, which will be used for robustness checks in our analysis. We asked subjects whether they had already bought Christmas gifts and how many gifts they intended to buy. They also had to state whether they had participated in Black Friday sales, and if so, how many products they had purchased on this day. Furthermore, subjects had to state whether they were confronted more frequently by solicitations and whether they donated more often, relative to the rest of the year.

### Results

**Fig 3:**
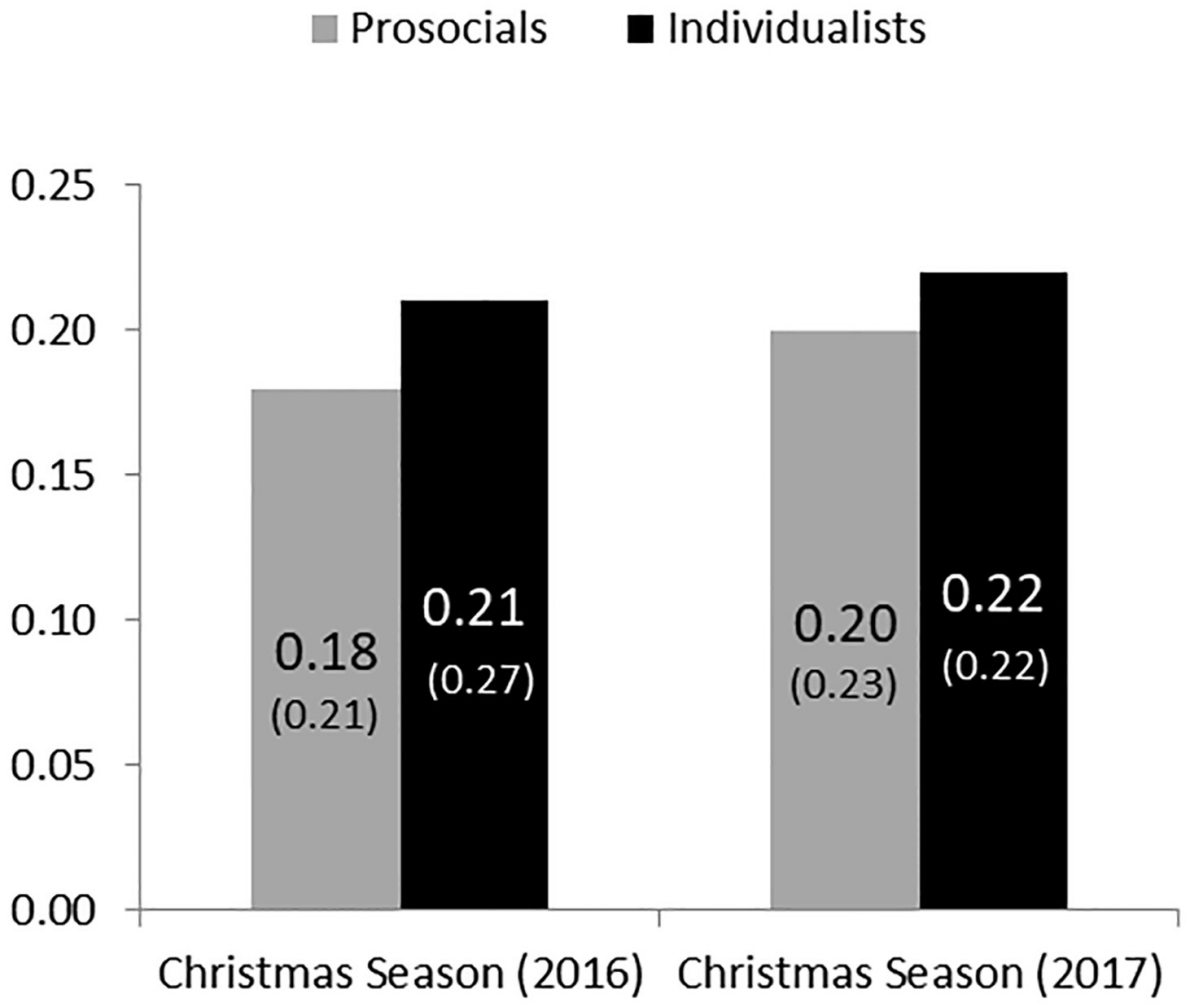
Percentage of donations across winter seasons conditioned on SVO. SD in parentheses.

We find that participants are very similar between the two winter samples in terms of their socio demographics (see Table B in S1 Appendix). Fig 3 compares the donation levels of prosocials and individualists in the Christmas season. The diagram distinguishes between the data of study 1 (left panel) and study 2 (right panel). After classifying our subjects on SVO types we have 66 subjects in our new data set. More precisely, we identify fifty prosocials and 16 individualists. Five subjects showed inconsistent choices and one subject was classified as competitive. These subjects were excluded from the further analyses, which we present below. It can be seen that average donations and the disaggregated data of prosocials and individualists are very similar across the two winter seasons.

This is confirmed by Kolmogorov-Smirnov tests, which find no differences between the two Christmas seasons for prosocials (*p* = .97) and individualists (*p* = .45). The replication of the Christmas season data in study 1 confirms the robustness of the crowding-out effect on charitable giving in winter. This is confirmed by regression analyses (see Table D in S1 Appendix, showing that the dummy of the Christmas-season 2016 is not significant.).

#### Analyses of potential drivers

Next, we focus on the relation between charitable giving and our two main variables, which presumably capture some of the specificity of the Christmas season *relative* to the rest of the year: *relative stress* and *relative savings*.

**Fig 4:**
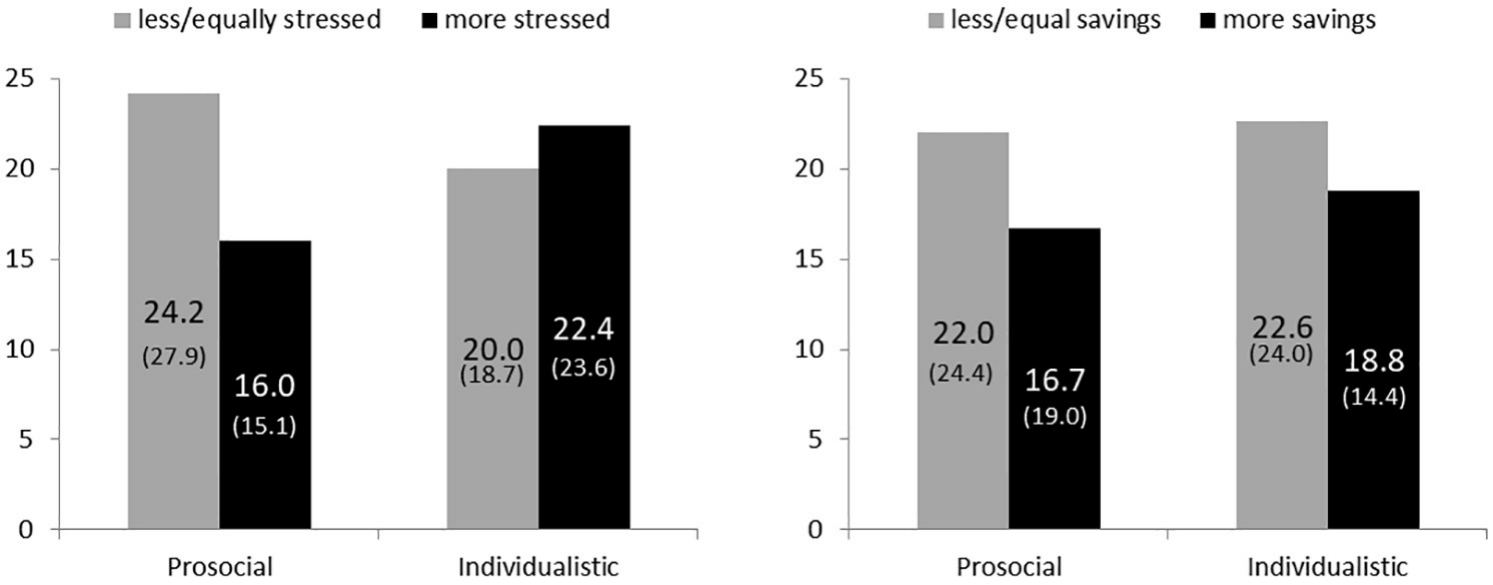
Percentage of donations conditioned on SVO types and self-reported levels of perceived stress and saving behavior.

The two measures of our questionnaire are negatively correlated with the donation level. That is, the more stress subjects feel relative to the rest of the year, the lower their giving. We also find that the more subjects save relative to the rest of the year, the lower their donated amount. The findings are confirmed by Pearson’s correlation coefficient tests (relative stress: *ρ* = − .27; *p* < .05; relative savings: *ρ* = − .22; *p* = .07). Fig 4 gives an overview of subjects’ donation levels conditioned on their SVO type and their relative stress (left panel) and saving levels (right panel).

For prosocials the diagram suggests that subjects who report increased levels of stress and savings (dark bars) donate less than subjects who report low levels (gray bars). Focusing on stress levels we find that this does not hold for individualistic subjects who always donate similar low levels. Moreover, we find that both types donate moderately less when reporting more savings. Interestingly, prosocials who report a low stress level (24.2%) in winter donate still less than prosocial subjects in summer. This shows that other channels, such as saving levels, may also be at work. This is indicated by the data. If we condition on prosocials who report neither higher stress levels nor higher saving levels, we find that this group donates the highest amount (26.3%).

Our results are supported by a three-way ANOVA test examining the effect of *relative stress*, *relative savings*, and *prosocial* (a dummy, which is positive for prosocials). We find a weakly significant general effect of *relative savings* (*F*(1, 66) = 2.20, *p* < .10) on donations. This is in line with our previous finding that generally all subjects who report more savings donate less. Whereas, *relative stress* (*F*(1, 66) = 0.47, *p* = .75), *prosocial* (*F*(1, 66) = .99, *p* = .32), the interaction of *relative savings* and *relative stress* (*F*(1, 66) = 1.04, *p* = .43), and the interaction between *relative savings* and *prosocial* (*F*(1, 66) = .30, *p* = .82) are not significant. The interaction of *relative stress* and *prosocial* is significant at the 5%-level (*F*(1, 66) = 3.70, *p* < .05). There is a moderate effect of the three-way interaction between *relative stress*, *relative savings*, and *prosocial* (*F*(1, 66) = 2.36, *p* = .10).

**Result 3**: *The higher prosocials’ reported stress level relative to the rest of the year, the lower their donations*.

Tobit regressions in Table E in S1 Appendix confirm the robustness of the effects of *relative stress* and *relative savings* on the donation levels in the Christmas season.

#### Robustness checks

We check the consistency of *relative stress* and *relative savings*. The fact that *relative stress* is related to subjects’ stress level, is supported by a positive correlation with the answers subjects gave in the Perceived Stress Questionnaire (PSQ) [29] (Pearson’s correlation coefficient, *ρ* = .28, *p* < .05). For *relative savings* we analyze to what extent this measure is consistent with other consumption-related measures in our questionnaire. Higher scores in *relative savings* ought to be negatively correlated with measures on actual or planned consumption. Information on actual or planned consumption is captured by the variables *bought a gift*, *planned # of Christmas gifts*, *Black Friday purchased*, and *Black Friday # of purchases*. *Bought gift* is a dummy, which is positive when subjects state that they had already bought a Christmas gift. *Planned # of Christmas gifts* is the self-reported number of intended gift purchases for Christmas. *Black Friday purchased* is a dummy, which is positive when subjects consumed on Black Friday. Whereas, *Black Friday # of purchases* is the self-reported number of product purchases on Black Friday. We find that *relative savings* is negatively correlated with all these measures, where the correlation is significant at the 5%-level for *bought gift* and *number of gifts*. This is confirmed by Pearson’s correlation coefficients (*ρ* = − .25, *p* < .05; *ρ* = −0.27, *p* < .05; *ρ* = − .17, *p* = .14, and *ρ* = − .09, *p* = .46).

Additionally, we analyze whether donations differ between subjects who reported that they were more often confronted with fundraising campaigns in the Christmas season relative to the rest of the year compared to subjects who did not state this. We do not find significant differences in the donation levels for these two groups (one-way ANOVA test, *F*(1, 66) = 0.14, *p* = .70). Donating more often in the Christmas season also has no effect than donating less often (one-way ANOVA test, *F*(1, 66) = 0.82, *p* = .37). Finally, we analyze whether subjects report being more stressed in the pre-Christmas period as suggested by other empirical studies [25]. We observe for the Christmas-season data that subjects are significantly more stressed relative to the rest of the year. This is documented by a significant one-sided t-test, which analyzes whether *relative stress* is higher than 3 (which questioned whether subjects were equally stressed between both seasons) (*t*(71) = 2.84, *p* < .01).

## Discussion and conclusion

We studied seasonal effects in charitable giving. Our controlled experiment shows that donations are significantly lower by about 30% in the Christmas season than in summer. We identify prosocial subjects and demonstrate that the effect is driven by them, as they are not only less likely to donate in winter, but also give significantly less if they do donate. To find some explanations we focused on two empirical particularities of the Christmas season, i.e., higher stress levels and higher consumption spending. We ran a second donation study with an extensive questionnaire, focusing on questions about perceived stressed levels, saving and consumption patterns during the Christmas season. First, the data indicate that the higher prosocials’ stress level around Christmas relative to the rest of the year, the lower the donations. This link between stress and donations is suggested by the combination of the following evidence from research in economics and psychology. As more stress is associated with a lower level of empathy [26], more stress may translate into lower donations by eroding subjects’ warm glow—the positive emotional feeling people get from helping others [30, 31, 32, 33]. Evidence by Declerck and Bogaert [34] suggests that a prosocial SVO correlates positively with the ability to adopt another person’s point of view. Davis et al. [35] find that higher scores in empathy questionnaires are positively correlated with prosocial behavior such as charitable giving. Second, we find that the higher the subjects’ self-reported relative savings, the lower their donations. The finding of a lower willingness to donate during Christmas is important, as it suggests that higher donations in the last quarter of a year may be primarily driven by the demand side, i.e., by aspects like tax incentives and intensive campaign activities. We are aware that the results on the possible channels are only indicative, as alternative explanations may exist. Nevertheless, we believe that the findings are promising as they are a good starting point for follow-up research to isolate these explanations when analyzing the consequences of emotional states on charitable giving.

We are aware that our studies have some limitations and therefore should be taken with a grain of salt. A first issue may be related to the experimental design. Although, our laboratory design increases control, allows for SVO elicitation, and guarantees a more homogeneous subject pool, problems may be related to external validity. First, our data focus mainly on students. Second, the setting may be perceived as artificial, since subjects come to the lab and receive a windfall endowment, which can be donated. This is different from real-life settings. Moreover, the lab data setting may cause selection effects, as subjects registering to experiments may come to the lab with the aim to earn money. Thus, it is possible that these people have different donation motives compared to people from outside the lab. Nevertheless, these potential issues are kept constant in the *whole* lab setting. Importantly, [36] demonstrate that experimental subjects are not different from people outside from the lab. In a large-scale survey study they compare the behavior of students, non-students, volunteers, and non-volunteers in dictator games. The authors conclude that self-selected students are an appropriate subject pool to study altruistic behavior. Another issue may be related to the SVO meassure, i.e., it is possible that the social-value orientation is not stable for all subjects. For instance, it may be that depending on the season when the SVO is measured some subjects may respond differently. In the most extreme case the consequence could be that we classify in the winter season some subjects as individualists, although they would have been classified as prosocials if the elicitation task was conducted in a summer season, and vice versa. This would bias the data and probably the correlation results of SVO types and donations. Nevertheless, we are quite confident that (even if it would be existent) this is not an issue in our data. The reason is simple, if our treatment effect (in Study 1) would be driven by a miss classification in SVO types we should observe the same average in donations between the seasons. However, this is not the case. Finally, one may ask whether in our questionnaire design (Study 2) further biases may play a role. For instance, some questionnaire studies are prone to a response bias such as the “social-desirability” bias. In this case, subjects would answer questions such that it will be viewed favorably by others. Although, this may be plausible in many questionnaire questions (such as questions on subjects’ helping or donation behavior) we do not see this issue arising for the set of questions we ask. The reason is that our questionnaire builds on questions about stress and consumption. Here, it is more than unclear how an answer should look like such that it is viewed favorable by others. In summary, although, there might be potential issues regarding external validity of our results, we believe that the results on the seasonal effects of donations are valuable, as they seem to be pretty robust. Of course, we are aware that there might be additional channels besides stress and consumption, which play a role for our main finding.

Despite these potential limitations, our results may contribute to fundraising strategies, as they provide interesting insights for the marketing, the timing, and the design of campaigns. Given the competition among many solicitations around Christmas and higher campaign costs caused by higher prices for print and media coverage, or for part-time employees, we suggest a careful cost-benefit analysis for running campaigns at this time. Contrary to conventional wisdom it might be more profitable to follow a counter-cyclic strategy and concentrate fund-raising activities outside gift-giving seasons. Our findings suggest, for example, that follow-up research it may be interesting to compare (donation) field experiments in relaxing environments (parks, spas) and stressful places such as train stations.

## Supporting information

S1 Appendix Appendix with Tables. (PDF)

S1 Text Translated experimental instructions. (PDF)

## References

[1] Center on Philanthropy at Indiana University, 2012. The 2012 bank of america study of high net worth philanthropy—issues driving charitable activities among wealthy households.

[2] MacLaughlin S., 2014. Charitable giving report: How nonprofit fundraising performed in 2014. Blackbaud, Charleston, 1-20.

[3] Cairns J., Slonim R., 2011 Substitution effects across charitable donations. Economics Letters 111, 173–175. 10.1016/j.econlet.2011.01.028

[4] Greenberg A.E., 2014 On the complementarity of prosocial norms: The case of restaurant tipping during the holidays. Journal of Economic Behavior & Organization 97, 103–112.

[5] Singh J., Teng N., Netessine S., 2019 Philanthropic campaigns and customer behavior: Field experiments on an online taxi booking platform. Management Science 65, 913–932. 10.1287/mnsc.2017.2887

[6] Hager M., 2002. An introduction to the overhead costs study: Methods and data, in: Annual Conference of the Association for Research on Nonprofit Organizations and Voluntary Action, Montreal Canada.

[7] Gneezy U., Keenan E.A., Gneezy A., 2014 Avoiding overhead aversion in charity. Science 346, 632–635. 10.1126/science.1253932 25359974

[8] Meer J., 2014 Effects of the price of charitable giving: Evidence from an online crowdfunding platform. Journal of Economic Behavior & Organization 103, 113–124.

[9] Eckel C.C., Grossman P.J., 2003 Rebate versus matching: Does how we subsidize charitable contributions matter? Journal of Public Economics 87, 681–701. 10.1016/S0047-2727(01)00094-9

[10] Ek C., 2017 Some causes are more equal than others? The effect of similarity on substitution in charitable giving. Journal of Economic Behavior & Organization 136, 45–62.

[11] Winking J., 2014 Anonymity versus privacy in the dictator game: Revealing donor decisions to recipients does not substantially impact donor behavior. PloS one, 9(12), e115419 10.1371/journal.pone.0115419 25532025 PMC4274055

[12] MacDonnell R., White K., 2015 How construals of money versus time impact consumer charitable giving. Journal of Consumer Research 42, 551–563.

[13] Raihani N.J., McAuliffe K., 2014 Dictator game giving: The importance of descriptive versus injunctive norms. PloS one 9, e113826 10.1371/journal.pone.0113826 25493945 PMC4262257

[14] Sonntag A., Zizzo D. J., 2015 On reminder effects, drop-outs and dominance: Evidence from an online experiment on charitable giving. PloS one, 10(8), e0134705 10.1371/journal.pone.0134705 26252524 PMC4529084

[15] Benz M., Meier S., 2008 Do people behave in experiments as in the field? Evidence from donations. Experimental Economics 11, 268–281. 10.1007/s10683-007-9192-y

[16] Gneezy U., Rustichini A., 2000 Pay enough or don’t pay at all. The Quarterly Journal of Economics 115, 791–810. 10.1162/003355300554917

[17] Duquette N.J., 2016 Do tax incentives affect charitable contributions? Evidence from public charities’ reported revenues. Journal of Public Economics 137, 51–69. 10.1016/j.jpubeco.2016.02.002

[18] Bekkers R., Wiepking P., 2011 A literature review of empirical studies of philanthropy: Eight mechanisms that drive charitable giving. Nonprofit and Voluntary Sector Quarterly 40, 924–973. 10.1177/0899764010380927

[19] Fischbacher U., 2007 z-Tree: Zurich toolbox for ready-made economic experiments. Experimental Economics 10, 171–178. 10.1007/s10683-006-9159-4

[20] Greiner B., 2015 Subject pool recruitment procedures: Organizing experiments with orsee. Journal of the Economic Science Association 1, 114–125. 10.1007/s40881-015-0004-4

[21] Gneezy U., Potters J., 1997 An experiment on risk taking and evaluation periods. Quarterly Journal of Economics 112, 631–645. 10.1162/003355397555217

[22] Van Lange P.A., Agnew C.R., Harinck F., Steemers G.E., 1997 From game theory to real life: How social value orientation affects willingness to sacrifice in ongoing close relationships. Journal of Personality and Social Psychology 73, 1330.

[23] Grosch K., Rau H. A., 2017 Gender differences in honesty: The role of social value orientation. Journal of Economic Psychology 62, 258–267. 10.1016/j.joep.2017.07.008

[24] Murphy R.O., Ackermann K.A., Handgraaf M., 2011 Measuring social value orientation. Judgment and Decision making 6, 771–781.

[25] Engel C., 2011 Dictator games: A meta study. Experimental Economics 14, 583–610. 10.1007/s10683-011-9283-7

[26] Park K.H., Kim D.H., Kim S.K., Yi Y.H., Jeong J.H., Chae J., et al, 2015 The relationships between empathy, stress and social support among medical students. International Journal of Medical Education 6, 103 10.5116/ijme.55e6.0d44 26342190 PMC4561553

[27] Kloner R.A., 2004 The “merry Christmas coronar” and “happy new year heart attack” phenomenon. Circulation 110, 3744–3745. 10.1161/01.CIR.0000151786.03797.18 15611386

[28] Waldfogel J., 1993 The deadweight loss of christmas. American Economic Review 83, 1328–1336.

[29] Levenstein S., Prantera C., Varvo V., Scribano M.L., Berto E., Luzi C., et al, 1993 Development of the perceived stress questionnaire: A new tool for psychosomatic research. Journal of Psychosomatic Research 37, 19–32. 10.1016/0022-3999(93)90120-5 8421257

[30] Andreoni J., 1989 Giving with impure altruism: Applications to charity and ricardian equivalence. Journal of Political Economy 97, 1447–1458. 10.1086/261662

[31] Andreoni J., 1990 Impure altruism and donations to public goods: A theory of warmglow giving. Economic Journal 100, 464–477. 10.2307/2234133

[32] Null C., 2011 Warm glow, information, and inefficient charitable giving. Journal of Public Economics 95, 455–465. 10.1016/j.jpubeco.2010.06.018

[33] Lilley A., Slonim R., 2014 The price of warm glow. Journal of Public Economics 114, 58–74. 10.1016/j.jpubeco.2013.12.004

[34] Declerck C.H., Bogaert S., 2008 Social value orientation: Related to empathy and the ability to read the mind in the eyes. The Journal of Social Psychology 148, 711–726. 10.3200/SOCP.148.6.711-726 19058659

[35] Davis M.H., Mitchell K.V., Hall J.A., Lothert J., Snapp T., Meyer M., 1999 Empathy, expectations, and situational preferences: Personality inuences on the decision to participate in volunteer helping behaviors. Journal of Personality 67, 469–503. 10.1111/1467-6494.00062 10483118

[36] Exadaktylos F., Espin A.M., Branas-Garza P., 2013 Experimental subjects are not different. Scientific reports 3, 1213 10.1038/srep01213 23429162 PMC3572448

