# Supplementary material for: Too cold for warm glow? Christmas-season effects in charitable giving
Source: PLoS One. 2019 May 22;14(5):e0215844. doi: 10.1371/journal.pone.0215844 (PMC6530824; doi:10.1371/journal.pone.0215844)
Supplement: S1 Appendix — (PDF) [file pone.0215844.s001.pdf]

S1 Appendix

Table A illustrates the randomization of subjects in the Christmas season 2016 and summer data (study 1). The table presents the data we used for the analysis, i.e., the SVO types who can not be classified are excluded.

Season	female (%)	age	econ (%)	prosocial (%)	risk
Christmas season 2016	.52 (.50)	24.24 (4.16)	.41 (.50)	.59 (.50)	44.51 (29.08)
summer	.56 (.50)	24.00 (3.03)	.44 (.50)	.67 (.47)	42.50 (28.67)
$p-value$	.62	.99	.76	.28	.55

Table A. Randomization of subjects with respect to their socio-demographics (SD in parentheses).

We present the $p-values$ of χ^2 tests for the variables: *female*, *econ*, and *prosocial*. *Female* is a dummy, which is positive for female students. *Econ* is a dummy, which is positive if subjects are either economic students or business economic students. We apply Kolmogorov-Smirnov tests for the variables: *age*, and *risk*. The data show no conspicuous differences in socio demographics, subjects' risk-taking behavior, and the fraction of classified prosocials between the two samples.

Table B compares subjects' demographics, their risk preferences, and the fraction of prosocials between the winter sample of the Christmas season 2016 (study 1) and the winter sample of the Christmas season 2017 (study 2) (SD in parentheses). The table presents the data we used for the analysis, i.e., the SVO types who can not be classified are excluded.

Season	female (%)	age	econ (%)	prosocial (%)	risk
Christmas season 2016	.52 (.50)	24.24 (4.16)	.41 (.50)	.59 (.50)	44.51 (29.08)
Christmas season 2017	.58 (.50)	23.65 (4.78)	.26 (.44)	.76 (.43)	45.09 (25.91)
$p-value$	.50	.27	.04	.03	.97

Table B. Overview of subjects' socio-demographics in our data (SD in parentheses).

We present the $p-values$ of χ^2 tests for the variables: *female*, *econ*, and *prosocial*. *Female* is a dummy, which is positive for female students. *Econ* is a dummy, which is positive if subjects are either economic students or business economic students. We apply Kolmogorov-Smirnov tests for the variables: *age*, and *risk*. It can be seen that the randomization was successful, i.e., subjects are very similar between the two winter samples in terms of their gender, age, and risk preferences. The only exceptions are that we had a lower fraction of econ students in study 2 and more prosocials. However, as our replication analysis shows, this has no effect on donation behavior in study 2, which is perfectly identical to study 1. This is also in line with the insight from study 1 of our paper that individualists and prosocials anyhow behave the same way in winter.

Table C presents Tobit regressions on donation levels, which are censored at 0 and 1. The regressions focus on the data of the summer and Christmas season of study 1. We include *Christmas season* (dummy, which is positive in winter); *prosocial* (dummy, which is positive for prosocials); the interaction of *Christmas season* $\times$ *prosocial*; *risk* (risk preference, meassured with our risk-elicitation task); *econ* (dummy, which is positive for economic and business economic students). Finally, we include subjects'

demographics. The regressions confirm our main findings: Subjects donate less in winter and prosocials generally give more. The donation levels are lower in winter because prosocials donate less at this time. All findings are robust when controlling for sociodemographics.

	donation level		
	(1)	(2)	(3)
Christmas season	-0.115**	0.135	0.138
	(0.057)	(0.096)	(0.096)
prosocial	0.118**	0.345***	0.343***
	(0.059)	(0.093)	(0.093)
Christmas × prosocial		-0.375***	–0.374***
		(0.118)	(0.118)
risk			0.001
			(0.001)
female			0.106*
			(0.060)
age			0.001
			(0.007)
econ			-0.032
			(0.058)
constant	0.145**	-0.013	-0.114
	(0.060)	(0.079)	(0.208)
obs.	156	156	156
R^2	0.052	0.111	0.134
Standard errors in parentheses			
*** p<0.01, ** p<0.05, * p<0.1			

Table C. Tobit regressions on subjects' donated amount in study 1.

Table D presents a Tobit regression on donation levels, which is censored at 0 and 1. The regression focuses on the two waves of the Christmas season data from 2016 (study 1) and 2017 (study 2). We include *Christmas season 2016* (dummy, which is positive for the data of 2016); *prosocial* (dummy, which is positive for prosocials); *risk* (risk preference, meassured with our risk-elicitation task); *econ* (dummy, which is positive for economic and business economic students). Finally we include subjects' demographics. The regressions confirm the findings reported in the main body of the paper. We find that *Christmas season 2016* is insignificant, emphasizing that the donation levels do not differ between the two waves of the Christmas seasons between study 1 and 2.

	donation level<br>(1)
Christmas season 2016	0.029
	(0.058)
prosocial	-0.034
	(0.060)
risk	0.000
	(0.001)
female	0.015
	(0.060)
age	-0.004
	(0.007)
econ	-0.118*
	(0.061)
constant	0.235
	(0.184)
obs.	158
R^2	0.031

Standard errors in parentheses
*** p<0.01, ** p<0.05, * p<0.1

Table D. Tobit regressions on subjects' donated amount in study 1.

Table E presents a Tobit regression on donation levels, which is censored at 0 and 1. The regression focuses on the Christmas season data from 2017 (study 2). In the regression we use the following regressors: *relative stress* (self-stated stress level relative to the rest of the year); *relative savings* (self-stated savings level relative to the rest of the year); *relative # of campaigns* (self-stated frequency of observed fundraising campaigns relative to the rest of the year); *Relative # donations* (self-stated number of supported solicitations relative to the rest of the year) *bought a gift* (dummy whether subjects had already bought a Christmas gift); *planned # of Christmas gifts* (stated number of Christmas gifts, subjects planned to buy in the current Christmas season); *Black Friday purchased* (dummy whether subjects did shopping on Black Friday); *Black Friday # of purchases* (number of purchases, which were made on Black Friday; *prosocial* (dummy, which is positive for prosocials); *risk* (risk preference, meassured with our risk-elicitation task); *econ* (dummy, which is positive for economic and business economic students). Finally, we include subjects' demographics. The regression confirms the robustness of our finding that more stress relative to the rest of the year and more savings relative to the rest of the year, lower donation levels in the winter data. Moreover, the savings level relative to the rest of the year also affects donations. *Econ* students donate lower levels.

	donation level<br>(1)
Relative stress	-0.057*
	(0.032)
Relative savings	-0.070**
	(0.033)
Relative # of campaigns	-0.016
	(0.050)
Relative # donations	-0.038
	(0.051)
Bought a gift	-0.022
	(0.077)
Planned # of Christmas gifts	-0.009
	(0.008)
Black Friday purchased	0.172
	(0.108)
Black Friday # of purchases	-0.055*
	(0.031)
prosocial	0.003
	(0.079)
risk	0.000
	(0.001)
female	0.022
	(0.077)
age	-0.014
	(0.009)
econ	-0.242***
	(0.081)
constant	0.617**
	(0.237)
obs.	66
Pseudo R^2	0.396

Standard errors in parentheses
*** p<0.01, ** p<0.05, * p<0.1

Table E. Tobit regressions on subjects' donated amount in study 2.
