# Supplementary material for: Too cold for warm glow? Christmas-season effects in charitable giving
Source: PLoS One. 2019 May 22;14(5):e0215844. doi: 10.1371/journal.pone.0215844 (PMC6530824; doi:10.1371/journal.pone.0215844)
Supplement: S1 Text — (PDF) [file pone.0215844.s002.pdf]

Instructions of the Experiment

Part 1

In part 1 you will find the following situation:

You have an endowment of 100 Taler which can be invested in a lottery.

The lottery wins or loses with a **probability of 50%.**

- If the lottery wins, your investment will be multiplied by 2,5.
- If the lotter loses, your investment will be lost.

<u>Please note:</u>

- You can only invest integers between 0 und 100 Taler.

If part 1 becomes payoff relevant, the computer will do a random draw which determines whether the lottery wins. The lottery will win with a probability of 50%.

In this case your payoff will be:

Not invested amount of the endowment + amount paid out by the lottery

You will receive the instructions for part two after you have made your decision in part one.

<u>Part 2</u>

In part two you have to decide on an allocation decision.

You have an endowment of 100 Taler. You are given the opportunity to donate Taler to the "German Red Cross." Therefore, you decide on the allocation of the endowment of **100 Taler** between **<u>you and the recipient</u>** ("German Red Cross").

Therefore, the following question will be displayed on the computer screen:

„Decide on the allocation of the 100 Taler between you and the German Red Cross."

I allocate to me: ______

I allocate to the German Red Cross: ______

<u>Please note:</u>

- You have to decide on the allocation of the entire *endowment* (100 Taler).
- You can only split integers (0-100 Taler).
- Your decision will remain anonymous after the end of the experiment.
- After the end of the experiment we will do an online transaction of the total sum of the donations to the German Red Cross. You are invited to stay and watch us doing the transaction.

If this part will be payoff relevant, then your payoff will equal the alloaction you dictated to you. At the same time the German Red Cross will exactly receive the amount you allocated to them.

You will receive the instructions for part three after you have made your decision in part two.

On-screen instructions of the SVO test (conducted after part three)

Imagine that another person was randomly matched with you. You do not know this person and you know that you will not meet this person in the future. You and the other person will make decisions by selecting one of the numbers 1, 2, or 3.

Your own decision will lead to points for you and the other person. At the same time, the decisions of the other person will also lead to points for you and for herself/himself. Each of these points is of value. The more points you receive, the better it is for you. The more points the other person receives the better it is for her/him.

In what follows, you will find an example of how these exercises will work:

	1	2	3
You will get	500	500	550
The other person will get	100	500	300

In this example the following holds: If you choose “1”, you would get 500 and the other person would get 100 points. If you choose “2”, you would get 500 points and the other person would also get 500. If you would choose “3”, you would get 550 points and the other person would get 300.

Thus, your decision and your own number of points also affects the other person’s number of points.

Before you make your decisions, bare in mind that there are no right and wrong answers. Just choose your preferred option.

Keep in mind that the points are of value, i.e., the more you get, the better. This also holds from the perspective of the other person. The more she/he gets, the better.

SVO test: Decision Sets

Decision set 1:

	1	2	3
You will get	480	540	480
The other person will get	80	280	480

Decision set 2:

	1	2	3
You will get	560	500	500
The other person will get	300	500	100

Decision set 3:

	1	2	3
You will get	520	520	580
The other person will get	520	120	320

Decision set 4:

	1	2	3
You will get	500	560	490
The other person will get	100	300	490

Decision set 5:

	1	2	3
You will get	560	500	490
The other person will get	300	500	90

Decision set 6:

	1	2	3
You will get	500	500	570
The other person will get	500	100	300

Decision set 7:

	1	2	3
You will get	510	560	510
The other person will get	510	300	110

Decision set 8:

	1	2	3
You will get	550	500	500
The other person will get	300	100	500

Decision set 9:

	1	2	3
You will get	480	490	540
The other person will get	100	490	300

Coding of the answers in correspondence to the social type

Prosocial choices are:

Decision set 1: choice 3; Decision set 2: choice 2; Decision set 3: choice 1; Decision set 4: choice 3; Decision set 5: choice 2; Decision set 6: choice 1; Decision set 7: choice 1; Decision set 8: choice 3; Decision set 9: choice 2.

Individualistic choices are:

Decision set 1: choice 2; Decision set 2: choice 1; Decision set 3: choice 3; Decision set 4: choice 2; Decision set 5: choice 1; Decision set 6: choice 3; Decision set 7: choice 2; Decision set 8: choice 1; Decision set 9: choice 3.

Competitive choices are:
Decision set 1: choice 1; Decision set 2: choice 3; Decision set 3: choice 2; Decision set 4: choice 1; Decision set 5: choice 3; Decision set 6: choice 2; Decision set 7: choice 3; Decision set 8: choice 2; Decision set 9: choice 1.

Note: Subjects can be classified as one of three social types, as a result of the most often chosen answer type. The classification is possible, if subjects gave at least five times the answer which corresponds to the same social type.

Questions of the: “Perceived Stress Questionnaire” (PSQ) Levenstein et al. (1993)

Please state for each statement, how often this was true during the last month (1 = almost never; 2 = sometimes; 3 = often; 4 = usually).

1. You feel rested.
2. You feel that too many demands are being made on you.
3. You are irritable or grouchy.
4. You have too many things to do.
5. You feel lonely or isolated.
6. You find yourself in situations of conflict.
7. You feel you’re doing things you really like.
8. You feel tired.
9. You fear you may not manage to attain your goals.
10. You feel calm.
11. You have too many decisions to make.
12. You feel frustrated.
13. You are full of energy.
14. You feel tense.
15. Your problems seem to be piling up.
16. You feel you’re in a hurry.
17. You feel safe and protected.
18. You have many worries.
19. You are under pressure from other people.
20. You feel discouraged.
21. You enjoy yourself.
22. You are afraid for the future.
23. You feel you’re doing things because you have to, not because you want to.
24. You feel criticized or judged.
25. You are lighthearted.
26. You feel mentally exhausted.
27. You have trouble relaxing.
28. You feel loaded down with responsibility.
29. You have enough time for yourself.
30. You feel under pressure from deadlines.

Score: 5 – chosen numbers for items: 1, 7, 10, 13, 17, 21, 25, 29.
Score: chosen numbers for all other items.

PSQ Index = (raw score – 30)/90.

Translation of the post-experimental questionnaire (second wave of the winter data: Christmas season 2017)

[Stress]

Since last month, do you perceive a higher level of stress compared to the rest of the year?

Select one from the 5 possible answers:

I perceive:

1. a much lower level of stress
2. a lower level level of stress
3. a similar level of stress
4. a higher level of stress
5. a much higher level of stress

[Savings]

Since last month, do you save more compared to the rest of the year?

Select one from the 5 possible answers:

I save:

1. much less
2. less
3. a similar level
4. more
5. much more

[Solicitation frequency

Since last month, do you perceive that there is a higher frequency of solicitations compared to the rest of the year?

Select one from the 5 possible answers:

I perceive:

1. a much lower solicitation frequency
2. a lower solicitation frequency
3. a similar frequency
4. a higher frequency
5. a much higher frequency

Since last month, do you support a higher number of solicitations compared to the rest of the year?

Select one from the 5 possible answers:

I support:

1. a much lower number
2. a lower number
3. a similar number
4. a higher number
5. a much higher number

[Consumption]

Have you already started buying Christmas gifts? Yes/No.

How many gifts are you planning to buy for Christmas? State number.

How many products have you purchased on Black Friday? State number.
